# Post-traumatic and iatrogenic silent sinus syndrome: a case series

**DOI:** 10.1007/s10006-025-01391-x

**Published:** 2025-05-22

**Authors:** E. M. Strabbing, O. Engin, M. A.J. Telleman, A. P. Nagtegaal, E. B. Wolvius

**Affiliations:** 1 Department of Oral and Maxillofacial Surgery, Special Dental Care and Orthodontics, University Medical Center Rotterdam, Dr. Molewaterplein 40, Rotterdam, 3015 GD the Netherlands; 2 Department of Ophthalmology, Erasmus Medical Centre, Rotterdam, The Netherlands; 3 Department of Otorhinolaryngology, Erasmus Medical Center, Rotterdam, The Netherlands

**Keywords:** Silent sinus syndrome, Imploding antrum, Orbital fracture, Iatrogenic, Maxillary sinus, Orbital floor reconstruction

## Abstract

**Objectives:**

Silent sinus syndrome (SSS) is a rare condition characterized by progressive maxillary sinus collapse, causing enophthalmos and hypoglobus without sinusitis symptoms. Secondary SSS arises from trauma or surgery disrupting mucociliary clearance. This study aims to analyze CT scan features, evaluate the timeline of SSS development, and identify contributing factors.

**Materials and methods:**

Patients diagnosed with secondary (post-traumatic or iatrogenic) SSS between January 2015 and January 2024 at the Erasmus Medical Center were reviewed. Characteristics from pre-SSS (T1) and post-SSS (T2) stages, management, and clinical outcomes were recorded. Data on patient demographics, symptoms, orthoptic findings, and the time interval between trauma or surgery and SSS onset were also collected.

**Results:**

Nine patients (six males and three females) met the inclusion criteria. The time from trauma or surgery to SSS onset ranged from one to thirty-six months, with a median of three months in the posttraumatic group. All patients presented with unilateral enophthalmos or hypoglobus; eight reported diplopia. Surgical management, including retrograde uncinectomy and orbital reconstruction, restored orbital anatomy and resolved symptoms.

**Conclusion:**

Secondary SSS is a rare but significant condition requiring early recognition to prevent severe cosmetic and functional complications.

**Clinical relevance:**

Secondary SSS should be considered when patients present with unexplained orbital changes following trauma or surgery. Regular follow-up is recommended, especially in patients with orbital trauma or surgery involving the inferomedial strut. Further studies are necessary to clarify risk factors associated with secondary SSS.

**Clinical trial number:**

Not applicable.

## Introduction

Silent sinus syndrome (SSS) is a rare disorder characterized by maxillary sinus atelectasis without sinus-related complaints. As the condition progresses, it can lead to orbital changes, resulting in spontaneous hypoglobus, enophthalmos, and/or diplopia. Primary SSS refers to cases of idiopathic origin with no history of previous sinus pathology, surgery, or trauma [1], while secondary SSS describes cases following facial trauma or iatrogenic events [2, 3]. The pathophysiology involves an obstruction of the ostiomeatal complex, leading to a cascade of hypoventilation, negative pressure, and subsequent collapse of the maxillary sinus walls [1, 4].

In cases of posttraumatic SSS, enophthalmos is not typically present at the time of the initial injury but develops as a delayed complication. The interval between trauma or surgery and the onset of secondary SSS can vary widely, ranging from three months to thirty years [2, 5]. However, many documented cases lack imaging immediately following trauma or surgery, making it difficult to confirm the prior absence of SSS [6]. To date, the risk factors predisposing the development of secondary SSS remain unclear. Early recognition of secondary SSS ensures timely treatment, minimizing the risk of severe cosmetic and functional sequelae and simplifying surgical management. This study presents a case series of secondary SSS from a tertiary referral hospital, aiming to study the computed tomography scan characteristics pre-and post-SSS, examine the timeline of the development of secondary SSS, and identify factors contributing to the onset of this condition.

## Materials and methods

This study was performed in line with the principles of the Declaration of Helsinki. Ethical approval was obtained (C-2014285) for the present retrospective cohort study. The case series included consecutive patients diagnosed with secondary (posttraumatic or iatrogenic) SSS between January 2015 and January 2024 at the Department of Oral & Maxillofacial Surgery of the Erasmus Medical Center, a tertiary referral center. The inclusion criteria were: (1) age > 18 years, (2) diagnosis of secondary SSS, and (3) availability of both a computed tomography (CT) or cone beam computed tomography (CBCT) scan at the time of trauma / initial surgery without signs of SSS (T1) and after confirmation of SSS (T2). Exclusion criteria included incomplete medical records and a history of sinusitis or other sinus pathology.

Patient demographics, symptoms, orthoptic findings, the time interval between the trauma or initial surgery, and the presentation of secondary SSS were collected. Two observers recorded CT or CBCT characteristics of both pre-SSS (T1) and post-SSS (T2), as well as the management and clinical outcomes. Due to the small cohort size, descriptive data were used to summarize the findings.

## Results

From 2015 to 2024, fourteen patients were diagnosed with secondary SSS. Nine of these cases met the inclusion criteria. The cohort consisted of six males and three females. Five cases were excluded due to incomplete records, one of whom also had a history sinus pathology.

Table 1 summarizes clinical data on age, fracture type, timing, orthoptic findings, and treatment. Seven patients had orbital fractures, all managed conservatively, while two had undergone external orbital decompression for Graves’ orbitopathy. The interval between trauma or surgery and the anamnestic onset of SSS ranged from one to thirty-six months, with a median of three months in the posttraumatic group. Notably, six patients developed secondary SSS within three months of trauma or iatrogenic intervention. The actual presentation at our tertiary center was later.

**Table 1:** Patients with secondary SSS

Patient	Sex (M/F)	Age	Type of fracture	Primary surgery	Interval onset secondary SSS (months)	Secondary SSS Symptoms*	Management SSS (ENT/Maxillofacial)	Single-staged
1	M	58	Orbital floor, zygoma,	No	15	H E D	Uncinectomy / PEEK	Yes
2	F	49	Orbital floor, medial wall	No	3	H E D	Claoué / PEEK	No
3	M	71	Orbital floor, medial wall	No	3	H E D	Claoué / PEEK	No
4	M	38	All orbital walls, frontal bone, zygoma	No	2	H E D	Uncinectomy / PEEK	Yes
5	M	68	Orbital floor, medial wall	No	1	H E D	Uncinectomy / PEEK	Yes
6	F	68	Orbital floor, lateral nasal wall, zygoma,	No	7	H E D	Uncinectomy / PEEK	Yes
7	M	34	Orbital floor, medial wall	No	3	H E D	Claoué / PEEK	Yes
8	M	37	Iatrogenic: medial wall + floor	Graves decompression (medial/medial floor)	3	H D	Uncinectomy/ decompression (medial/lateral)	Yes
9	F	47	Iatrogenic: medial wall + floor	Graves decompression (medial wall/floor)	36	H E	Claoué	N/A

Note: H = hypoglobus, E = enophthalmos, D = diplopia. PEEK refers to a patient-specific polyetheretherketone orbital implant, N/A = Not applicable

All patients presented with unilateral enophthalmos and/or hypoglobus, with diplopia reported in eight of nine cases. The most common orthoptic findings were hypotropia of the affected side (strabismus with one eye deviated downwards when both eyes are open) and limited upgaze, causing vertical diplopia. Orbital floor fractures were the most prevalent, with some extending to adjacent structures such as the medial orbital wall, zygoma, and lateral nasal wall.

In the posttraumatic group, five cases were managed using a single-stage approach, which included retrograde uncinectomy or Claoué (inferior meatal antrostomy; used when the orbit obstructed the middle meatus), combined with orbital reconstruction using a patient-specific polyetheretherketone (PEEK) implant. The choice for PEEK implants was based on the surgeon’s preference and experience with the material. A two-stage approach was performed in two cases, involving initial maxillary sinus aeration followed by orbital reconstruction in a second procedure.

Of the two patients with iatrogenic SSS, one patient was treated with an infundibulotomy combined with further orbital decompression (orbital reconstruction was not indicated). The other underwent a Claoué procedure without orbital reconstruction, as she was not bothered by the mildly altered position of her eye.

Patients were followed for one year at the orbital outpatient clinic, where they were assessed by a multidisciplinary team, including a maxillofacial surgeon, ophthalmologist, and orthoptist. Each patient also had a single postoperative evaluation by an ENT specialist. When nasal endoscopy confirmed a well-aerated maxillary sinus with healed mucosa, the risk of recurrence was considered negligible. In all patients, hypotropia and diplopia were resolved after surgery.

**Fig. 1:**
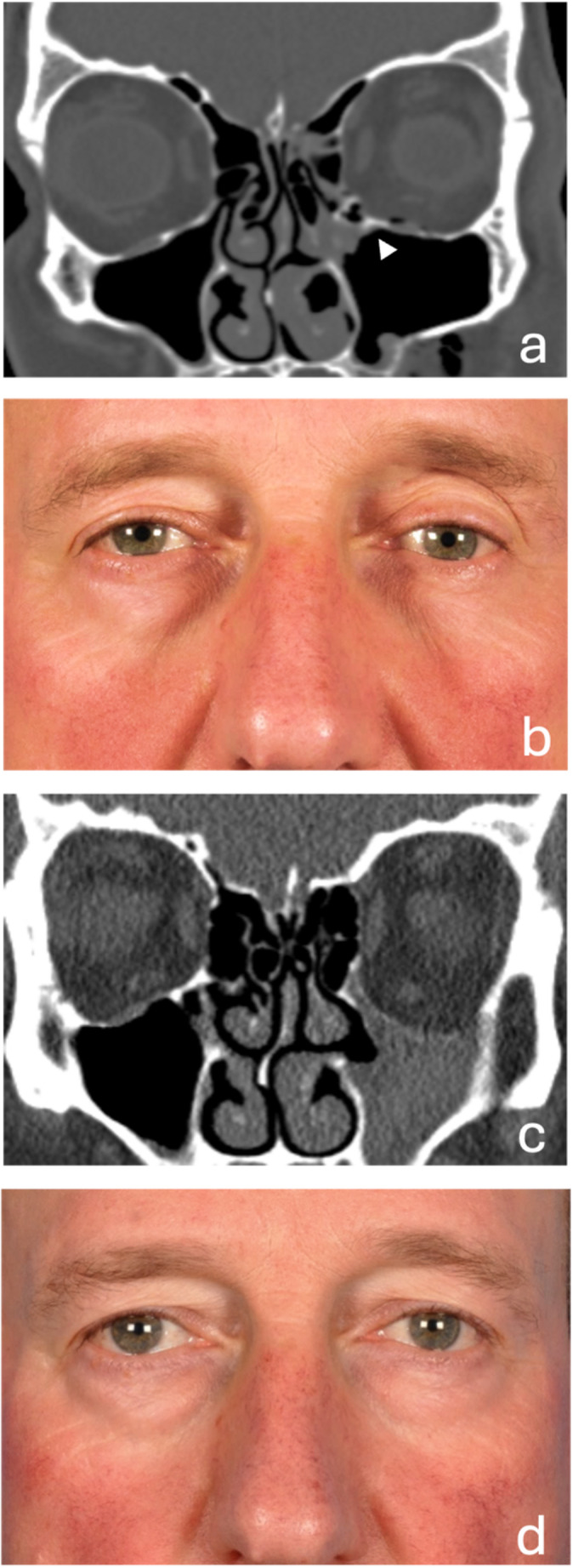
Posttraumatic secondary silent sinus syndrome (SSS). Note: A 58-year-old male patient following facial trauma. (**A**) Coronal CT at T1 (time of trauma). The scan shows a left zygomatic fracture involving the orbital floor and maxillary ostium region (arrow). Opacification of the infundibulum is noted, but no other signs of sinus pathology are evident at this stage. (**B**) Clinical photograph, twenty-one months post-trauma. There is a visible asymmetry in the upper eyelid sulci and light reflection on both corneas, consistent with enophthalmos and hypoglobus in the left eye. The patient reports diplopia on upgaze, which emerged six months after the trauma. (**C**) Coronal CT scan at T2. Signs of secondary SSS are evident, including complete opacification of the left maxillary sinus, lateralization of the left uncinate process, and depression of the orbital floor. (**D**) Clinical photograph, eleven months post-surgery. Following endoscopic retrograde uncinectomy and orbital reconstruction with a patient-specific PEEK implant. Diplopia is no longer present, and the left eye has been restored to a normal globe position, confirmed by Hertel measurements (12-118-12)

**Fig. 2:**
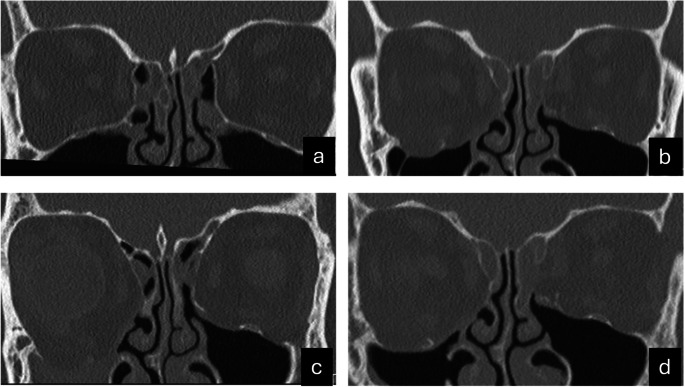
Iatrogenic secondary silent sinus syndrome (SSS). Note: Coronal CT images of a 51-year-old woman with Graves orbitopathy. (**A**). Before orbital decompression, the maxillary sinus shows no pathology. (**B**). After external orbital decompression of the right medial wall and orbital floor with involvement of the inferomedial strut (T1), adequate aeration of the right maxillary sinus is observed. Note the opacification of the ethmoid sinuses, most likely related to the decompression procedure. (**C**) Despite an uneventful postoperative course, progressive right enophthalmos and hypoglobus were noted, though the patient did not experience any symptoms of diplopia. The CT scan shows signs of secondary SSS (T2), including opacification of the right maxillary sinus, further depression of the orbital floor, and lateral deviation of the uncinate process. (**D**). Twelve months post-surgery, the CT scan shows a recovery of maxillary sinus aeration. Due to obstruction of the middle meatus caused by orbital prolapse, a Claoué procedure (inferior meatal antrostomy) was performed to restore function.

Table 2 shows the anatomical structures involved in pre-SSS (T1), identifying fractures of the orbital floor in all cases and involvement of the inferomedial strut and/or maxillary sinus ostium in seven cases at T1. Table 3 outlines the CT or CBCT SSS-related characteristics at T2 for all patients. The most consistent findings included complete opacification of the affected maxillary sinus, lateral deviation of the uncinate process, and increased ipsilateral orbital volume, indicative of SSS-related changes. Figures 1 and 2 present two representative cases—one posttraumatic and one post-surgical—to illustrate the diagnostic and therapeutic management of secondary SSS.

**Table 2:** Anatomical structures involved in pre-SSS (T1)

Patient	Pre-SSS scans (T1)
1	Inferomedial strut, ostium, orbital floor
2	Inferomedial strut, ostium, orbital floor
3	Inferomedial strut, ostium, orbital floor
4	Orbital floor, medial wall
5	Orbital floor, medial wall
6	Inferomedial strut, ostium, orbital floor
7	Inferomedial strut, ostium, orbital floor
8	Inferomedial strut, ostium, orbital floor
9	Inferomedial strut, ostium, orbital floor, medial wall

Note. The table shows that most patients with secondary SSS exhibited involvement of multiple anatomical structures in pre-SSS (T1) scans, including the inferomedial strut, maxillary sinus Ostium, and orbital floor. The inferomedial strut, also known as the transitional zone, is a key anatomical structure defined as a bony buttress located at the junction of the medial and inferior orbital walls

**Table 3:** Post-SSS (T2) features on CT scans

Patient	Complete opacification of the affected sinus	Lateral deviation of the uncinate process	Reduction of the volume of the maxillary antrum with retraction of the maxillary walls	Increase in ipsilateral orbital volume	Demineralization of the sinus walls	Expanded retroantral fat
1	**X**	**X**	**X**	**X**	**X**	**X**
2	**X**	**X**	**X**	**X**	**X**	
3	**X**	**X**		**X**	**X**	
4	**X**	**X**		**X**	**X**	
5	**X**	**X**		**X**	**X**	
6	**X**	**X**	**X**	**X**	**X**	
7		**X**	**X**	**X**	**X**	
8	**X**	**X**	**X**	**X**		
9	**X**	**X**	**X**	**X**	**X**	**X**

Note. Post-SSS (T2) scans showed consistent features across patients, with an ‘X’ marked for each feature identified in the scan

## Discussion

This case series aims to analyze computed tomography scan features, examine the timeline of SSS development, and identify factors contributing to its onset. Our findings reveal that most secondary SSS cases develop in the first three months after trauma or surgery. A notable radiological feature in patients who develop SSS after trauma or orbital surgery is the presence of combined orbital fractures involving the orbital floor and medial wall, including the inferomedial strut.

This cohort reflects a predominance of male patients (66,7%), likely due to their greater exposure to physical injuries, as noted in previous trauma research [7]. The time between trauma or surgery and the onset of SSS symptoms ranged from one to thirty-six months, consistent with existing literature [2, 6]. As a tertiary referral center, we expect there will be some delay in referral, suggesting that clinical signs of SSS may have been present for some time before patients had access to specialized care. Additionally, it remains debatable whether SSS occurring many years later can still be attributed to the initial trauma. The median time to presentation in posttraumatic patients is three months. These findings emphasize the importance of follow-up after trauma, even for simple fractures not requiring surgery, particularly those close to the maxillary ostium. Imaging is essential when unexpected changes in globe position and/or an increased Hertel difference are observed. Early detection and intervention are critical to prevent progressive functional and cosmetic deficits.

In contrast with other studies [5, 8], diplopia was a presenting symptom in all but one patient, consistently appearing in conjunction with enophthalmos and hypoglobus. Patients with diplopia exhibited more pronounced hypoglobus and enophthalmos than those without it. Hypotropia, limited upgaze, and vertical diplopia were seen in most patients. Given that diplopia tends to develop as orbital dystopia progresses, this pattern likely reflects a delayed diagnosis of secondary SSS in our cohort. This delay may be attributed to the tertiary referral nature of our department, as some patients had already consulted other specialists before being referred to us.

Previous studies on secondary SSS lack information on the posttraumatic/postsurgical imaging showing no signs of SSS. The absence of immediate post-traumatic imaging in published cases makes it challenging to pinpoint the exact moment the syndrome begins to develop, raising questions about the underlying mechanisms and predisposing factors. In our group, the diagnosis of secondary SSS was confirmed by post-traumatic or post-surgical CT scans showing no signs of sinus pathology. Further imaging analysis revealed that fractures involving the orbital floor, inferomedial strut, and maxillary sinus ostium were consistently identified in pre-SSS imaging. These findings align with the theory that obstruction of the maxillary ostium leads to progressive negative pressure, sinus collapse, and orbital floor displacement, contributing to the development of SSS [8, 9].

Although involvement of the maxillary ostium is commonly associated with orbital floor fractures or combined fractures of the floor and medial wall, the development of posttraumatic SSS is still rarely reported [2, 10]. This suggests that a disruption of normal sinus function and anatomy is likely a critical factor in the development of this pathology. However, the underlying factors that trigger the development of SSS in cases of orbital trauma remain unclear. The authors hypothesize that the current trend toward a more conservative approach in orbital fracture treatment [11, 12], may inadvertently increase the risk of secondary SSS. Despite this, the rarity of this condition does not warrant surgical intervention in the absence of clinical indications for orbital fracture repair. Acknowledging the risk of secondary SSS and advising patients to seek evaluation if they experience symptoms or concerns is essential for timely management.

In our cohort, all patients underwent surgical treatment for their SSS, with (retrograde) uncinectomy and orbital reconstruction being the most frequently performed procedures. The decision to proceed with orbital reconstruction was based on the patient’s functional and aesthetic complaints. A single-staged approach was mostly used in cases of significant enophthalmos and diplopia. In contrast, the two posttraumatic cases treated solely by restoring natural sinus ventilation showed no improvement in diplopia or eye position. As a result, a second surgery with orbital reconstruction was performed, leading to the resolution of the symptoms. This suggests that although restoring sinus ventilation is important, it may not be sufficient in cases with clear orbital symptoms.

For cases with mild enophthalmos without diplopia, a two-step surgical approach may be considered, as some patients do not require orbital floor reconstruction or may opt out of further surgery if the altered eye position is not functionally or cosmetically disturbing [5, 8]. Although there are no strict criteria for selecting between a one- or two-stage procedure, our experience suggests that a single-stage approach is often effective. SSS is not a primarily infectious process, and in our series, no postoperative infections occurred with PEEK implants. Therefore, delaying orbital reconstruction solely out of concern for infection may not be necessary. Ultimately, all patients achieved satisfactory outcomes, with restored orbital anatomy and resolution of symptoms.

This study is limited by its small cohort size, which prevented statistical analysis and limited the generalizability of the findings. Further research is needed to identify specific factors contributing to secondary SSS development. Prospective studies incorporating routine post-trauma or post-surgical follow-up would provide valuable data on the early detection and progression of the syndrome. Additionally, exploring the role of individual patient factors, such as anatomical variations, sinus mucosal health, or genetic predispositions, could enhance our understanding of why some patients develop SSS while others do not.

## Conclusion

SSS is a rare disorder of maxillary sinus atelectasis occurring without sinus-related complaints, caused by ostium occlusion. This leads to shrinking of the maxillary sinus, orbital floor depression, and enophthalmos. A key radiological feature in patients developing SSS after trauma / orbital surgery is the presence of an orbital floor fracture with involvement of the inferomedial strut, typically treated conservatively. Follow-up is recommended for this patient group, as early identification and intervention in case of unexpected symptoms can help reduce the severity of secondary SSS and its associated complications.

## Data Availability

No datasets were generated or analysed during the current study.
